# 4-Acetyl-3-[2-(eth­oxy­carbon­yl)phen­yl]sydnone

**DOI:** 10.1107/S1600536813027207

**Published:** 2013-10-19

**Authors:** David Grossie, Leanna Harrison, Kenneth Turnbull

**Affiliations:** a Department of Chemistry, Wright State University, Dayton, OH 45435, USA

## Abstract

Sydnones, which contain a mesoionic five-membered heterocyclic ring, are more stable if synthesized with an aromatic substutuent at the N3 position. In the title compound {sys­tematic name: 4-acetyl-3-[2-(eth­oxy­carbon­yl)phen­yl]-1,2,3-oxa­diazol-3-ylium-5-olate}, C_13_H_12_N_2_O_5_, the aromatic substitutent is 2-(eth­oxy­carbon­yl)phenyl. Intra- and inter­molecular hydrogen bonds are observed. The inter­planar angle between the sydnone and benzene rings is 71.94 (8)°. π-ring⋯carbon­yl inter­actions of 3.2038 (16) Å arise between the sydnone ring and a symmetry-related C=O group.

## Related literature

For more information on the sydnone family of compounds, see: Ohta & Kato (1969 ▶). For synthesis and structure information, see: Grossie & Turnbull (1992 ▶); Grossie *et al.* (2001 ▶, 2007 ▶); Hope & Thiessen (1969 ▶); Hodson & Turnbull (1985 ▶); Riddle *et al.* (2004*a* ▶,*b* ▶,*c* ▶); Hanley *et al.* (1976 ▶). For stability of the temperature controller used for the data collection, see: Cosier & Glazer (1986 ▶).
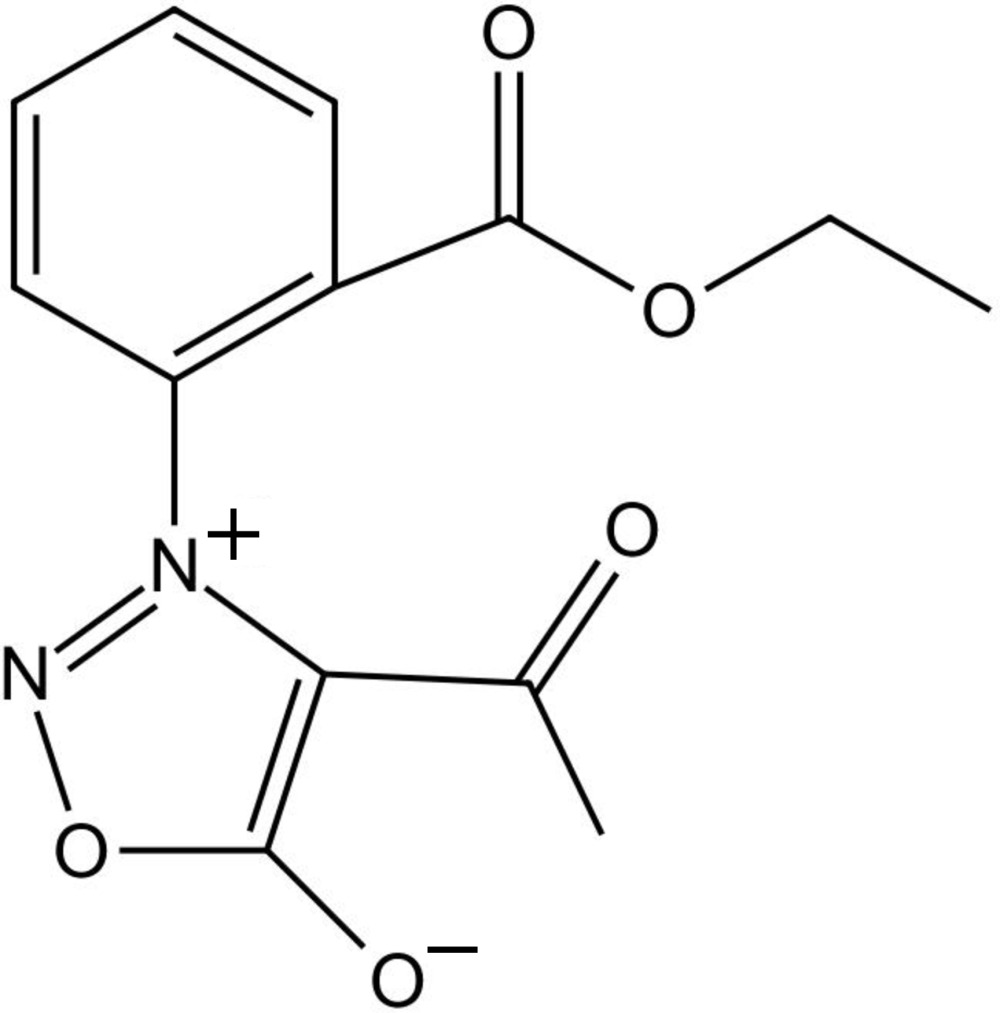


## Experimental

### 

#### Crystal data

C_13_H_12_N_2_O_5_
*M* *_r_* = 276.25
Monoclinic, 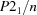

*a* = 11.353 (3) Å
*b* = 8.093 (2) Å
*c* = 14.607 (4) Å
β = 112.582 (4)°
*V* = 1239.1 (6) Å^3^
*Z* = 4
Mo *K*α radiation
μ = 0.12 mm^−1^
*T* = 173 K
0.22 × 0.20 × 0.17 mm

#### Data collection

Bruker Kappa APEXII diffractometer
Absorption correction: multi-scan (*SADABS*; Sheldrick, 1996 ▶) *T*_min_ = 0.90, *T*_max_ = 0.98
13826 measured reflections
3718 independent reflections
2783 reflections with *I* > 2σ(*I*)
*R*_int_ = 0.038

#### Refinement

*R*[*F* ^2^ > 2σ(*F* ^2^)] = 0.050
*wR*(*F* ^2^) = 0.121
*S* = 0.96
3718 reflections
181 parameters
H-atom parameters constrained
Δρ_max_ = 0.49 e Å^−3^
Δρ_min_ = −0.38 e Å^−3^

### 

Data collection: *APEX2* (Bruker, 2006 ▶); cell refinement: *APEX2*; data reduction: *APEX2*; program(s) used to solve structure: *SIR92* (Altomare *et al.*, 1994 ▶); program(s) used to refine structure: *CRYSTALS* (Betteridge *et al.*, 2003 ▶); molecular graphics: *CAMERON* (Watkin *et al.*, 1996 ▶); software used to prepare material for publication: *CRYSTALS*.

## Supplementary Material

Crystal structure: contains datablock(s) global, I. DOI: 10.1107/S1600536813027207/gg2123sup1.cif

Structure factors: contains datablock(s) I. DOI: 10.1107/S1600536813027207/gg2123Isup2.hkl

Supplementary material file. DOI: 10.1107/S1600536813027207/gg2123Isup3.cml

Additional supplementary materials:  crystallographic information; 3D view; checkCIF report

## Figures and Tables

**Table 1:** Hydrogen-bond geometry (Å, °)

*D*—H⋯*A*	*D*—H	H⋯*A*	*D*⋯*A*	*D*—H⋯*A*
C36—H361⋯O5^i^	0.95	2.51	3.253 (2)	136
C40—H401⋯O41^ii^	0.97	2.46	3.116 (2)	124
C42—H423⋯O5	0.96	2.51	3.065 (2)	117

Symmetry codes: (i) 
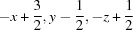
; (ii) 
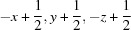
.

**Fig. 1.**
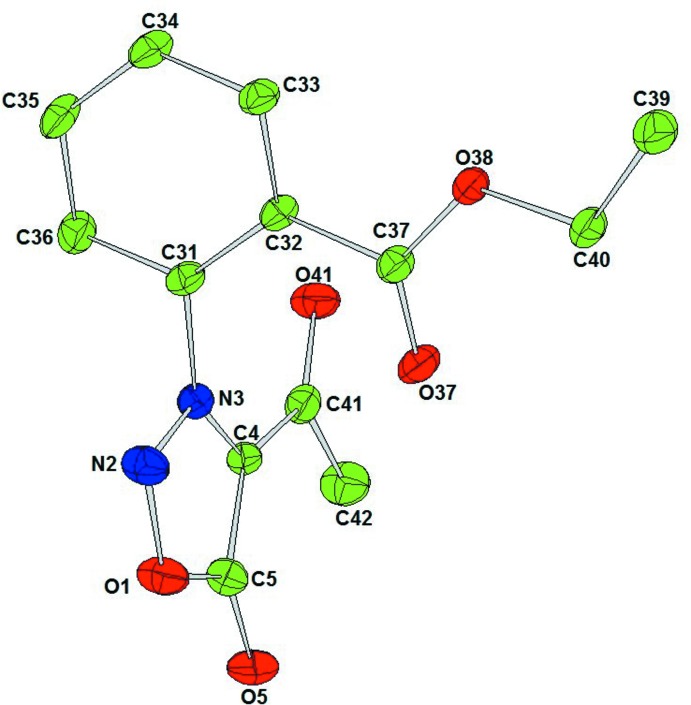
The title compound with displacement ellipsoids drawn at the 50% probability level. H atoms are shown as spheres of arbitary radius.

## supplementary crystallographic information

### 1. Comment

The bond distances and angles were within expected values. The sydnone ring (O1 – C5) and phenyl ring (C31 – C36) of the structure are planar as expected, with all deviations less than 0.1 Å. The angle between the planes of the sydnone (O1 – C5) and phenyl ring (C31 – C36) is 71.94 (8)°. π-atom interactions are seen between the sydnone ring and a symmetry-related O(5) with a distance of 3.2038 (16) Å. Numerous short intra and inter-molecular contacts are noted within the structure. The potential H bonds in the structure are tabulated below.

### 2. Experimental

4-Acetyl-3-(2-ethoxycarbonylphenyl)sydnone was synthesized in 47% yield by heating 3-[2-(ethoxycarbonyl)phenyl]sydnone, acetic anhydride (5 eq), bismuth trifluoromethanesulfonate (25 mole %), and lithium perchlorate (25 mole %) in acetonitrile (2 ml) in a sealed tube at 140°C for 5 h.

### 3. Refinement

The H atoms were all located in a difference map, but those attached to carbon atoms were repositioned geometrically. The H atoms were initially refined with soft restraints on the bond lengths and angles to regularize their geometry (C—H in the range 0.93–0.98, N—H in the range 0.86–0.89 N—H to 0.86 O—H = 0.82 Å) and *U*_iso_(H) (in the range 1.2–1.5 times *U*_eq_ of the parent atom), after which the positions were refined with riding constraints.

### Crystal data

**Table:**

C_13_H_12_N_2_O_5_	*F*(000) = 576
*M**_r_* = 276.25	*D*_x_ = 1.481 Mg m^−^^3^
Monoclinic, *P*2_1_/*n*	Mo *K*α radiation, λ = 0.71073 Å
Hall symbol: -P 2yn	Cell parameters from 2783 reflections
*a* = 11.353 (3) Å	θ = 6–60°
*b* = 8.093 (2) Å	µ = 0.12 mm^−^^1^
*c* = 14.607 (4) Å	*T* = 173 K
β = 112.582 (4)°	Block, colourless
*V* = 1239.1 (6) Å^3^	0.22 × 0.20 × 0.17 mm
*Z* = 4	

### Data collection

**Table:**

Bruker Kappa APEXII diffractometer	2783 reflections with *I* > 2σ(*I*)
Graphite monochromator	*R*_int_ = 0.038
ω scans	θ_max_ = 30.6°, θ_min_ = 2.0°
Absorption correction: multi-scan (*SADABS*; Sheldrick, 1996)	*h* = −15→15
*T*_min_ = 0.90, *T*_max_ = 0.98	*k* = −11→11
13826 measured reflections	*l* = −20→20
3718 independent reflections	

### Refinement

**Table:**

Refinement on *F*^2^	Primary atom site location: structure-invariant direct methods
Least-squares matrix: full	Secondary atom site location: difference Fourier map
*R*[*F*^2^ > 2σ(*F*^2^)] = 0.050	Hydrogen site location: inferred from neighbouring sites
*wR*(*F*^2^) = 0.121	H-atom parameters constrained
*S* = 0.96	Method = Modified Sheldrick *w* = 1/[σ^2^(*F*^2^) + ( 0.05*P*)^2^ + 0.68*P*], where *P* = (max(*F*_o_^2^,0) + 2*F*_c_^2^)/3
3718 reflections	(Δ/σ)_max_ = 0.0001892
181 parameters	Δρ_max_ = 0.49 e Å^−^^3^
0 restraints	Δρ_min_ = −0.38 e Å^−^^3^

### Special details

**Table:**

Experimental. The crystal was placed in the cold stream of an Oxford Cryosystems open-flow nitrogen cryostat (Cosier & Glazer, 1986) with a nominal stability of 0.1 K.
Geometry. Least-squares planes (*x*,*y*,*z* in crystal coordinates) and deviations from them (* indicates atom used to define plane)Sydnone:O1—C50.6390 (6) *x* - 0.6043 (6) *y* - 0.4760 (7) *z* = -0.206 (6) (11)* 0.005 (1) O1 * 0.003 (1) N2 * -0.010 (1) N3 * 0.012 (1) C4 *- 0.010 (2) C50.1671 (6) *x* - 0.0840 (6) *y* + 0.9824 (1) *z* = 5.3188 (17)Attached phenyl ring: C31–36* -0.003 (1) C31 * 0.004 (1) C32 * -0.002 (1) C33 * -0.001 (2) C34 * 0.002 (2) C35 * 0.000 (2) C36Angle to previous plane (with approximate e.s.d.) = 71.94 (8)

### Fractional atomic coordinates and isotropic or equivalent isotropic displacement parameters (Å^2^)

**Table:**

	*x*	*y*	*z*	*U*_iso_*/*U*_eq_	
O41	0.40593 (10)	0.21460 (14)	0.17007 (8)	0.0279	
C41	0.49814 (14)	0.30225 (18)	0.18948 (11)	0.0208	
C4	0.60889 (13)	0.28357 (17)	0.28115 (10)	0.0175	
N3	0.62198 (11)	0.16639 (15)	0.35007 (9)	0.0189	
N2	0.72926 (12)	0.16842 (18)	0.42560 (10)	0.0265	
O1	0.79450 (10)	0.29896 (15)	0.40892 (9)	0.0296	
C5	0.72170 (14)	0.37830 (19)	0.31878 (11)	0.0222	
O5	0.76294 (11)	0.49824 (14)	0.29284 (9)	0.0293	
C31	0.53744 (14)	0.03347 (17)	0.35081 (11)	0.0190	
C36	0.57526 (15)	−0.12260 (18)	0.33667 (12)	0.0242	
C35	0.50160 (16)	−0.25590 (18)	0.34078 (12)	0.0267	
C34	0.39295 (15)	−0.23188 (19)	0.35861 (12)	0.0253	
C33	0.35629 (14)	−0.07463 (18)	0.37287 (11)	0.0218	
C32	0.42817 (14)	0.06187 (17)	0.36981 (10)	0.0191	
C37	0.39015 (14)	0.23053 (18)	0.38796 (11)	0.0203	
O38	0.28355 (10)	0.22771 (13)	0.40506 (8)	0.0243	
C40	0.23638 (15)	0.38788 (19)	0.42045 (13)	0.0264	
C39	0.12669 (18)	0.3580 (2)	0.45073 (15)	0.0354	
O37	0.44855 (11)	0.35433 (13)	0.38822 (9)	0.0296	
C42	0.50663 (16)	0.4341 (2)	0.12175 (12)	0.0296	
H361	0.6494	−0.1359	0.3224	0.0288*	
H351	0.5251	−0.3639	0.3321	0.0328*	
H341	0.3441	−0.3213	0.3614	0.0302*	
H331	0.2821	−0.0586	0.3855	0.0256*	
H402	0.3053	0.4420	0.4736	0.0308*	
H401	0.2089	0.4494	0.3586	0.0317*	
H393	0.0942	0.4636	0.4613	0.0530*	
H392	0.1546	0.2974	0.5114	0.0523*	
H391	0.0588	0.2989	0.3991	0.0514*	
H423	0.5461	0.5316	0.1577	0.0481*	
H422	0.5591	0.3955	0.0874	0.0479*	
H421	0.4258	0.4594	0.0745	0.0459*	

### Atomic displacement parameters (Å^2^)

**Table:**

	*U*^11^	*U*^22^	*U*^33^	*U*^12^	*U*^13^	*U*^23^
O41	0.0226 (5)	0.0250 (6)	0.0288 (6)	−0.0055 (4)	0.0018 (4)	0.0058 (5)
C41	0.0232 (7)	0.0167 (6)	0.0234 (7)	0.0017 (5)	0.0099 (6)	0.0009 (5)
C4	0.0181 (6)	0.0150 (6)	0.0211 (6)	−0.0005 (5)	0.0094 (5)	0.0016 (5)
N3	0.0186 (5)	0.0172 (5)	0.0208 (6)	0.0000 (4)	0.0075 (5)	0.0015 (5)
N2	0.0213 (6)	0.0296 (7)	0.0245 (6)	−0.0027 (5)	0.0043 (5)	0.0058 (5)
O1	0.0226 (5)	0.0320 (6)	0.0296 (6)	−0.0068 (5)	0.0048 (5)	0.0055 (5)
C5	0.0211 (7)	0.0237 (7)	0.0231 (7)	−0.0016 (5)	0.0100 (6)	0.0006 (6)
O5	0.0289 (6)	0.0269 (6)	0.0348 (6)	−0.0090 (5)	0.0153 (5)	0.0016 (5)
C31	0.0221 (7)	0.0149 (6)	0.0195 (6)	−0.0020 (5)	0.0075 (5)	0.0025 (5)
C36	0.0276 (7)	0.0192 (7)	0.0280 (8)	0.0029 (6)	0.0130 (6)	0.0031 (6)
C35	0.0367 (9)	0.0134 (6)	0.0319 (8)	0.0022 (6)	0.0154 (7)	0.0020 (6)
C34	0.0322 (8)	0.0155 (7)	0.0297 (8)	−0.0040 (6)	0.0135 (7)	0.0011 (6)
C33	0.0252 (7)	0.0172 (6)	0.0249 (7)	−0.0023 (5)	0.0118 (6)	0.0020 (6)
C32	0.0239 (7)	0.0145 (6)	0.0194 (7)	−0.0010 (5)	0.0089 (6)	0.0014 (5)
C37	0.0245 (7)	0.0166 (6)	0.0221 (7)	−0.0004 (5)	0.0115 (6)	0.0003 (5)
O38	0.0268 (5)	0.0162 (5)	0.0356 (6)	−0.0010 (4)	0.0185 (5)	−0.0018 (4)
C40	0.0307 (8)	0.0163 (7)	0.0380 (9)	0.0002 (6)	0.0195 (7)	−0.0032 (6)
C39	0.0397 (10)	0.0244 (8)	0.0538 (11)	−0.0024 (7)	0.0311 (9)	−0.0041 (8)
O37	0.0375 (6)	0.0160 (5)	0.0455 (7)	−0.0042 (4)	0.0274 (6)	−0.0040 (5)
C42	0.0307 (8)	0.0287 (8)	0.0266 (8)	−0.0010 (6)	0.0081 (7)	0.0090 (6)

### Geometric parameters (Å, º)

**Table:**

O41—C41	1.2047 (18)	C34—H341	0.922
C41—C4	1.450 (2)	C33—C32	1.3842 (19)
C41—C42	1.483 (2)	C33—H331	0.938
C4—N3	1.3490 (18)	C32—C37	1.486 (2)
C4—C5	1.410 (2)	C37—O38	1.3266 (18)
N3—N2	1.2918 (17)	C37—O37	1.2006 (18)
N3—C31	1.4445 (18)	O38—C40	1.4527 (18)
N2—O1	1.3648 (17)	C40—C39	1.493 (2)
O1—C5	1.4118 (19)	C40—H402	0.969
C5—O5	1.2006 (18)	C40—H401	0.972
C31—C36	1.375 (2)	C39—H393	0.967
C31—C32	1.390 (2)	C39—H392	0.955
C36—C35	1.380 (2)	C39—H391	0.972
C36—H361	0.948	C42—H423	0.958
C35—C34	1.369 (2)	C42—H422	0.966
C35—H351	0.937	C42—H421	0.935
C34—C33	1.379 (2)		
			
O41—C41—C4	121.51 (13)	C34—C33—H331	120.2
O41—C41—C42	122.68 (14)	C32—C33—H331	118.7
C4—C41—C42	115.81 (13)	C31—C32—C33	117.18 (13)
C41—C4—N3	124.76 (12)	C31—C32—C37	122.01 (12)
C41—C4—C5	129.68 (13)	C33—C32—C37	120.81 (13)
N3—C4—C5	105.55 (12)	C32—C37—O38	111.49 (12)
C4—N3—N2	115.20 (12)	C32—C37—O37	124.70 (13)
C4—N3—C31	130.25 (12)	O38—C37—O37	123.80 (13)
N2—N3—C31	114.47 (12)	C37—O38—C40	115.53 (11)
N3—N2—O1	104.81 (12)	O38—C40—C39	107.47 (13)
N2—O1—C5	110.80 (11)	O38—C40—H402	107.1
O1—C5—C4	103.58 (12)	C39—C40—H402	110.2
O1—C5—O5	120.17 (14)	O38—C40—H401	108.6
C4—C5—O5	136.25 (15)	C39—C40—H401	110.6
N3—C31—C36	115.86 (13)	H402—C40—H401	112.7
N3—C31—C32	121.65 (13)	C40—C39—H393	108.5
C36—C31—C32	122.40 (13)	C40—C39—H392	109.9
C31—C36—C35	118.82 (14)	H393—C39—H392	108.5
C31—C36—H361	119.5	C40—C39—H391	110.7
C35—C36—H361	121.7	H393—C39—H391	108.8
C36—C35—C34	120.17 (14)	H392—C39—H391	110.5
C36—C35—H351	120.9	C41—C42—H423	111.3
C34—C35—H351	118.9	C41—C42—H422	108.9
C35—C34—C33	120.39 (14)	H423—C42—H422	107.4
C35—C34—H341	119.9	C41—C42—H421	110.7
C33—C34—H341	119.7	H423—C42—H421	110.1
C34—C33—C32	121.04 (14)	H422—C42—H421	108.3

### Hydrogen-bond geometry (Å, º)

**Table:**

*D*—H···*A*	*D*—H	H···*A*	*D*···*A*	*D*—H···*A*
C36—H361···O5^i^	0.95	2.51	3.253 (2)	136
C40—H401···O41^ii^	0.97	2.46	3.116 (2)	124
C33—H331···O38	0.94	2.33	2.681 (2)	101
C42—H423···O5	0.96	2.51	3.065 (2)	117

Symmetry codes: (i) −*x*+3/2, *y*−1/2, −*z*+1/2; (ii) −*x*+1/2, *y*+1/2, −*z*+1/2.

## References

[bb1] Altomare, A., Cascarano, G., Giacovazzo, C., Guagliardi, A., Burla, M. C., Polidori, G. & Camalli, M. (1994). *J. Appl. Cryst.* **27**, 435.

[bb2] Betteridge, P. W., Carruthers, J. R., Cooper, R. I., Prout, K. & Watkin, D. J. (2003). *J. Appl. Cryst.* **36**, 1487.

[bb3] Bruker (2006). *APEX2* Bruker AXS Inc., Madison, Wisconsin, USA.

[bb4] Cosier, J. & Glazer, A. M. (1986). *J. Appl. Cryst.* **19**, 105–107.

[bb5] Grossie, D. A., Sun, L. & Turnbull, K. (2007). *Acta Cryst.* E**63**, o2042–o2043.

[bb6] Grossie, D. A. & Turnbull, K. (1992). *Acta Cryst.* C**48**, 377–379.

[bb7] Grossie, D. A., Turnbull, K. & Krein, D. M. (2001). *Acta Cryst.* E**57**, o985–o987.

[bb8] Hanley, R. N., Ollis, W. D. & Ramsden, C. A. (1976). *J. Chem. Soc. Chem. Commun.* **9**, 306–307.

[bb9] Hodson, S. J. & Turnbull, K. (1985). *J. Heterocycl. Chem.* **22**, 1223–1227.

[bb10] Hope, H. & Thiessen, W. E. (1969). *Acta Cryst.* B**25**, 1237–1247.

[bb11] Ohta, M. & Kato, H. (1969). *Nonbenzenoid Aromatics*, edited by J. P. Snyder, pp. 117–248. New York: Academic Press.

[bb12] Riddle, G. B., Grossie, D. A. & Turnbull, K. (2004*a*). *Acta Cryst.* E**60**, o977–o978.

[bb13] Riddle, G. B., Grossie, D. A. & Turnbull, K. (2004*b*). *Acta Cryst.* E**60**, o1568–o1570.

[bb14] Riddle, G. B., Grossie, D. A. & Turnbull, K. (2004*c*). *Acta Cryst.* E**60**, o258–o259.

[bb15] Sheldrick, G. M. (1996). *SADABS* University of Göttingen, Germany.

[bb16] Watkin, D. J., Prout, C. K. & Pearce, L. J. (1996). *CAMERON* Chemical Crystallography Laboratory, Oxford, England.

